# The association of cholesterol absorption gene Numb polymorphism with Coronary Artery Disease among Han Chinese and Uighur Chinese in Xinjiang, China

**DOI:** 10.1186/s12944-015-0102-6

**Published:** 2015-09-29

**Authors:** Mayila Abudoukelimu, Zhen-Yan Fu, Ailifeire Maimaiti, Yi-Tong Ma, Minawaer Abudu, Qing Zhu, Dilare Adi, Yi-Ning Yang, Xiao-Mei Li, Xiang Xie, Fen Liu, Bang-Dang Chen

**Affiliations:** Department of Cardiology, The First Affiliated Hospital of Xinjiang Medical University, Urumqi, 830001 People’s Republic of China; Xinjiang Key Laboratory of Cardiovascular Disease Research, Urumqi, 830001 People’s Republic of China; The First Affiliated Hospital of Xinjiang Medical University, Urumqi, 830001 People’s Republic of China; Present address: The First Affiliated Hospital of Xinjiang Medical University, Li Yu Shan South Road 137, Urumqi, 830001 People’s Republic of China; Present address: Xinjiang Key Laboratory of Cardiovascular Disease Research, Li Yu Shan South Road 137, Urumqi, 830001 People’s Republic of China

**Keywords:** Coronary artery disease, Case–control study, Genetic polymorphism, Numb

## Abstract

**Background:**

Hypercholesterolemia is a major risk factor for coronary artery disease (CAD). As Numb is an important regulating factor for intestinal cholesterol absorption and plasma cholesterol level, the aim of the present study is to assess the association between human Numb gene polymorphism and CAD among Han and Uighur Chinese.

**Methods:**

We have conducted two independent case–control studies in Han Chinese (384 CAD patients and 433 controls) and Uighur Chinese (506 CAD patients and 351 controls) subjects. All subjects were genotyped for four kinds of SNPs (rs12435797, rs2108552, rs1019075 and rs17781919) and SNP is used as a genetic marker for human Numb gene. Genotyping was undertaken using TaqMan SNP genotyping assay, and the subjects’ ethnicity and gender were considered in the analysis.

**Results:**

We found that rs2108552 was associated with CAD in the dominant model (CC vs CG + GG) for the total Han Chinese population (*n* = 200) and Han Chinese males (*n* = 115) (*P* = 0.004 and *P* = 0.001, respectively). The difference remained statistically significant after multivariate adjustment (total: OR = 1.687, *P* = 0.004; male: OR = 1.498, *P* = 0.006). Further, for the total (*n* = 817) and male (*n* = 490) Han Chinese, the frequency of the haplotype (T-C-T-C) was significantly higher in the CAD patients than in the controls (*P* = 0.004 and *P* = 0.002), and the frequency of the haplotype (G-G-T-C) was significantly lower in the CAD patients than in the control subjects (*P* = 0.013, *P* = 0.007). In addition, for the total (*n* = 857) and male (*n* = 582) Uighur Chinese, we observed that rs12435797 was associated with CAD in an additive and recessive model (*P* = 0.021 and *P* = 0.009; *P* = 0.048 and *P* = 0.034). However, the difference did not remain statistically significant after multivariate adjustment. The overall distribution of rs2108552, rs1019075 and rs17781919 genotypes, alleles and the frequency of the haplotype established by four SNPs showed no significant difference between CAD patients and control subjects in the total, male and female Uighur Chinese.

**Conclusions:**

The results of this study indicate that CC genotype of rs2108552 and T-C-T-C haplotypes in Numb gene is a possible risk genetic marker and G allele and G-G-T-C haplotypes is a possible protective genetic marker for CAD in male Han Chinese.

## Introduction

Cholesterol is an important structural component of cell membranes, and a precursor for bile acids, vitamin D, and steroid hormone [1]. Accumulative studies have established that the high concentration of blood cholesterol level is closely related to an increase risk of Coronary Artery Disease (CAD), which is becoming increasingly prevalent and is a leading cause of death in developed countries [2–4]. In addition, to environmental factors, plasma cholesterol level is also influenced by genetic factors such as single nucleotide polymorphisms (SNPs) [5].

Cholesterol homeostasis is mainly maintained by denovo synthesis, intestinal absorption, and biliary and fecal excretion in the human body. Studies have shown that elevated plasma cholesterol level can be reduced by inhibiting exogenous cholesterol absorption, and therefore prevent the development of atherosclerotic cardiovascular disease [6–9].

There are several genes such as that encoding Niemann-Pick C1-Like 1 (NPC1L1) protein [10, 11] that are involved with intestinal cholesterol pathway [12–17]. Further, the role of NPC1L1 in cholesterol abosorption is mainly attributed to the clathrin-dependent endocytosis [18, 19]. Pei Shan Li et al. [20] has revealed that there exists an interaction between clathrin adaptor Numb and NPC1L1 during the regulation of cholesterol absorption. They have also found that Numb recognizes the particular endocytic motif (YVNXXF) in C-terminal of NPC1L1, finishes the internalization by recruiting clathrin/AP2, and transports cholesterol to endocytic recycling compartment (ERC) through microfilaments.

Numb is a member of clathrin-associated sorting proteins, and combines with several other endocytic proteins [21–24]. The gene is located on chromosome 14q24.3 that encodes two important domains. One of them is phosphotyrosine-binding (PTB) domain and another one is proline-rich region (PRR) domain [25–28]. Among four different protein isoforms of Numb which are p71 (PTBSPRRL), p65 (PTBSPRRS), p72 (PTBLPRRL) and p66 (PTBLPRRS), only isoform p71 and p65 with shorter PTB domains directly combine with NPC1L1 and stimulate NPC1L1 endocytosis.

Jian Wei et al. [29] confirmed that there was a remarkable correlation between Numb polymorphism G595D (rs17781919) and low concentration of LDL-C among humans. Besides, Jian Wei et al. believe that rs17781919 influences Numb activity during NPC1L1 internalization and reduces cholesterol absorption. In addition, it is generally accepted that a low concentration of LDL-C in plasma is an important factor for delaying the development of atherosclerotic cardiovascular disease.

No case–control studies have been conducted to assess the association between Numb gene and CAD. Therefore, the aim of this study is to clarify the relationship between polymorphism of gene Numb and CAD in Han Chinese and Uighur Chinese.

## Materials and methods

### Subjects

All patients with CAD and control subjects were recruited from The First Affiliated Hospital of Xinjiang Medical University from January 2007 to December 2013. Han Chinese patients and Uighur Chinese patients were studied independently. CAD group included 384 Han Chinese patients and 506 Uighur Chinese patients, and the control group included 433 Han Chinese and 351 Uighur Chinese. CAD was defined as the presence of at least one significant coronary artery stenosis with more than 50 % luminal diameter on coronary angiography. Control subjects also underwent a coronary angiogram, and were confirmed to be free of coronary artery stenosis. Moreover, these subjects did not show clinical or electrocardiogram evidence of myocardial infarction (MI) or CAD as described previously [30, 31]. However, some control subjects had cardiovascular risk factors such as essential hypertension (EH), diabetes mellitus (DM) or hyperlipidemia, but they had no history of MI or CAD. All information and data regarding EH, DM, hyperlipidemia and smoking were collected from all study subjects, and they were matched between two CAD and control cohorts individually. Hypertension was established if patients were on antihypertensive medication or if the mean of 3 measurements of systolic blood pressure (SBP) > 140 mmHg or diastolic blood pressure (DBP) >90 mmHg, respectively. Diabetes mellitus was diagnosed according to the criteria of the American Diabetes Association. Hyperlipidemia was defined as a total plasma cholesterol > 6.22 mmol or plasma triglycerides >2.26 mmol and /or the current use of lipid-lowering drugs with an established diagnosis of hyperlipidemia. Smoking status was dichotomized as smokers (current and ex-smokers) or non-smokers [32]. CAD patients and control subjects were free of impaired malignancy, connective tissue disease, renal function, valvular disease and chronic inflammatory disease.

### Anthropometric and biochemical variables measurement

Height and body weight were measured as described previously [33], and body mass index (BMI) was calculated by dividing the weight in kilogram to the square of height in meter. Further, WHO Asia-Pacific Area criterion- BMI ≥25 kg/m^2^ was used to define obesity as described previously [33]. Finally, blood urea nitrogen (BUN), creatinine (Cr), uric acid, total cholesterol (TC), triglyceride (TG), low density lipoprotein-cholesterol (LDL-C), and high density lipoprotein-cholesterol (HDL-C) were measured by using chemical analysis equipment (Dimension AR/AVL Clinical Chemistry System, Newark, NJ) in Clinical Laboratory Department of The First Affiliated Hospital of Xinjiang Medical University [34, 35]. Friedewald formula was used in the calculation of very low density lipoprotein (VLDL) [36] as shown in the following: VLDL = 1/5 of plasma TG level (mmol/L). TG < 4 mmol/L is required for the formula as a cut off and the reference value for VLDL is 0.11–0.34 in our study.

### Ethical approval of the study protocol

All participants have given their written informed consent and explicit permission for DNA analysis as well as for the collection of relevant clinical data. This study was approved by the Ethics Committee of The First Affiliated Hospital of Xinjiang Medical University (Urumqi, China) and was conducted by strictly following the requirements of the Declaration of Helsinki.

### SNP selection

**Fig. 1:**
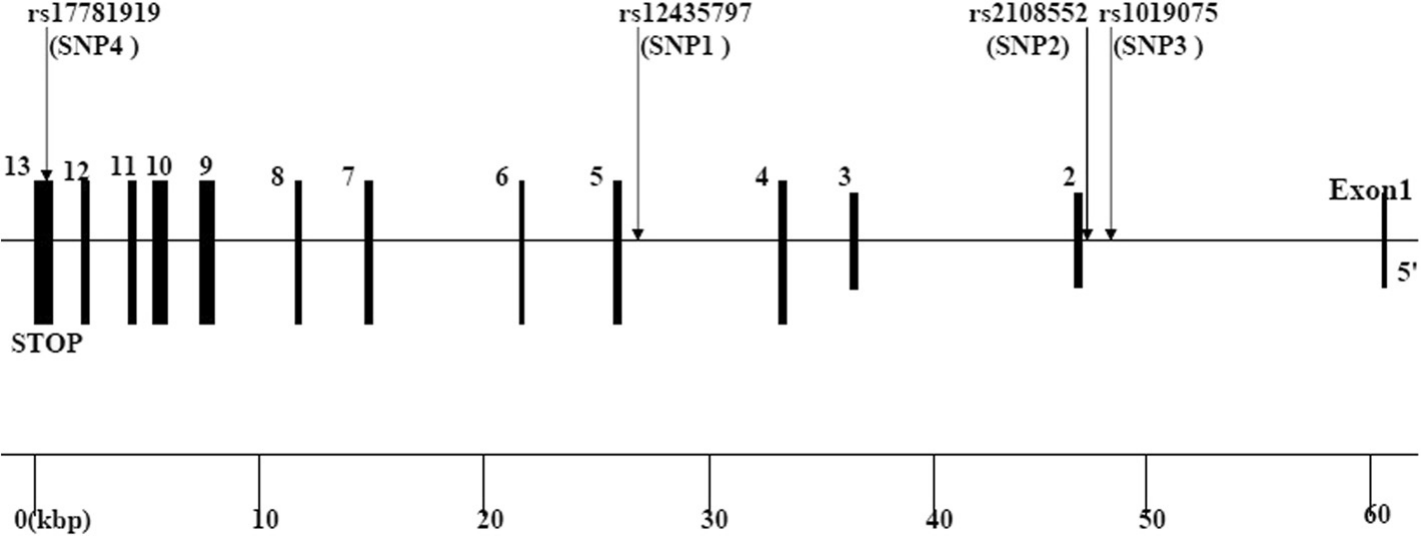
Structure of the human Numb gene. This gene consists of 13 exons separated by 12 introns. Boxes indicate exons, and lines indicate introns and intergenic regions. Arrows mark the locations of polymorphisms

The human Numb gene consists of 651 amino acids and is located on chromosome 14q24.3.It contains 13 exons which are further separated by 12 introns. There are 3781 different kinds of SNPs of human Numb gene as listed in theNational Center for Biotechnology Information SNP database (http://www.ncbi.nlm.nih.gov/snp). For this current study, we have screened the HapMap phase I& II database and Haploview 4.0 software for the tag SNPs of Numb gene and selected three SNPs (rs2108552, rs12435797, and rs1019075). Each of them conforms to the standards of minor allele frequency (MAF) ≥0.1 and linkage disequilibrium patterns with r^2^ ≥ 0.8 as a cut-off [30]. Meanwhile, we also included rs17781919 from the Numb gene which was associated with LDL-C [29]. rs2108552, rs12435797, and rs1019075 are located in intron. rs17781919 is located in exon13, and had a non-synonymous substitution amino acid change, which is defined by an C-to-T nucleotide substitution that leads to an exchange of Glycine by Aspartic acid at amino acid position 595. The position of the four SNPs (rs12435797, rs2108552, rs1019075 and rs17781919) was by order of increasing distance from the Numb gene 5`end (Fig. 1).

### Genotyping

Blood samples were taken from all participants by using anticoagulant ethylene diamine tetraacetic acid (EDTA) tube, and standard phenol-chloroform method was used to extract genomic DNA from peripheral leukocytes [37]. Samples were stored at −80 °C until use. DNA was diluted to 50 ng/μL concentration for real time PCR. Further, genotyping was undertaken by using TaqMan® SNP Genotyping Assay (Applied Biosystems) [38]. The probes and primers used in TaqMan®SNP Genotyping Assays (ABI) were selected according to the information on ABI website (http://www.appliedbiosystems.com). Allele-specific fluorogenic probes were hybridised to the template in the first step of the 5′ nuclease assay. During the polymerase chain reaction (PCR), the 5′ nuclease activity of the Taq polymerase made it possible for discrimination. In addition, the probes include a 3′ minor groove binding group that hybridises to single-stranded targets and has greater sequence specificity when compared to the original DNA probes. This reduces nonspecific probe hybridization which leads to low background fluorescence for the 5′ nuclease PCR assay (TaqMan; Applied Biosystems). Cleavage results in the increased emission of a reporter dye. Two unlabeled PCR primers and two allele-specific probes were required for each 5′ nuclease assay. At the 5′ end, each probe is labeled with two reporter dyes. In the present study, VIC and FAM were used as the reporter dyes. Finally, PCR amplification was performed by using 1 μL of DNA, 3 μL of TaqMan Universal Master Mix, 1.95 μL ddH_2_O, and 0.05 μL TaqMan SNP Genotyping Assay Mix (40×) containing a 331.2nM final concentration of primers and a 73.6nM final concentration of the probes. In addition, thermal cycling conditions for PCR amplification were 95 °C for 10 min, 45 cycles of 95 °C for 10 s and 60 °C for 1 min. Moreover, thermal cycling was undertaken by using Applied Biosystems7900HT Standard Real-Time PCR System, and all 96 well plates were read according to Sequence Detection Systems (SDS) automation controller software v2.3 (ABI).

### Statistical analysis

All statistical analyses were performed via using SPSS 16.0 software for Windows (SPSS Institute, Chicago, IL, USA). All continuous variables were expressed via mean ± standard deviation and the differences in continuous variables between CAD patients and control subjects were compared by using an independent-sample *T*-test. Further, Chi square analysis was used to test the deviations of genotype distribution from the Hardy-Weinberg equilibrium and to determine the differences of allele or genotype frequencies between patients and controls. Finally, logistic regression analyses with effect ratios (odds ratio [OR] and 95 % CI [confidence interval]) were used to assess the contribution of the major risk factors.

Based on the genotype data of the genetic variations, we performed haplotype-based case–control analyses using the expectation maximization algorithm and the software SNPAlyze version 3.2 (Dynacom, Yokohama, Japan). The SHEsis platform was used to verify reliability for SNPAlyze [38]. In the haplotype-based case–control analysis, haplotypes with a frequency of < 0.03 were excluded. The frequency distribution of the haplotypes was calculated by performing a permutation test using the bootstrap method. *P* < 0.05 was considered to statistically significant.

For the analysis to succeed, all variants should be located in one haplotype block, which is indicated by a large |D'| value between each SNP (near 1). When the r^2^ values are large (near 1) for the pairwise variants, one variant is not needed. The LD analysis was performed using four SNP pairs. We used |D'| values of > 0.25 to assign SNP locations to one haplotype block. SNPs with an r^2^ value > 0.5 were selected as tagged.

Dominant (common allele homozygotes coded as 1 and heterozygotes and recessive allele homozygotes as 2); recessive (recessive allele homozygotes as 1, common allele homozygotes and heterozygotes coded as 2) and additive (heterozygotes as 1, recessive allele homozygotes and common allele homozygotes coded as 2) models were considered in the Chi square analysis.

## Results

### Characteristics of included subjects

The two study cohorts included a total of 1674 subjects (1072 male and 602 female). There were 384 Han Chinese CAD patients (270 male and 114 female) and 506 Uighur Chinese CAD patients (369 male and 137 female). 433 Han (220 male and 213 female) and 351 Uighur individuals (213 male and 138 female) were included as controls. All subjects were recruited from out-patient and in-patient departments of The First Affiliated Hospital of Xinjiang Medical University from January 2007 to December 2013.

### Results of outcome measures

1. Clinical and metabolic characteristics of the subjects
  i. In Han Chinese, BMI (Body Mass Index), plasma concentration of glucose (Glu), triglyceride (TG), total cholesterol (TC), low density lipoprotein-cholesterol (LDL-C), very low density lipoprotein (VLDL),prevalence of DM, hyperlipidemia and smoking was statistically higher in patients with CAD when compared to control subjects (Table 1).
  ii. In Uighur Chinese, BMI, plasma concentration of Glu, BUN, TC, TG, LDL-C, VLDL, HDL-C, prevalence of EH, DM, hyperlipidemia and smoking was significantly higher in CAD patients in comparison to controls (Table 2).

**Table 1:** Characteristics of subjects (Han Chinese)

	Total			Male			Female		
	CAD patients	Control subjects	*P* Value	CAD patients	Control subjects	*P* Value	CAD patients	Control subjects	*P* Value
Number(n)	384	433		270	220		114	213	
Age(years)	59.65 ± 0.501	58.91 ± 0.470	0.284	58.13 ± 0.563	58.86 ± 0.618	0.427	58.25 ± 0.714	58.95 ± 0.589	0.335
BMI(kg/m^2^)	26.03 ± 0.167	25.22 ± 0.151	0.000^*^	26.34 ± 0.194	25.33 ± 0.212	0.010^*^	26.29 ± 0.315	25.12 ± 0.232	0.000^*^
Glu(mmol/L)	6.58 ± 0.162	5.48 ± 0.111	0.000^*^	6.22 ± 0.211	6.07 ± 0.196	0.000^*^	6.18 ± 0.200	5.77 ± 0.146	0.000^*^
BUN(mmol/L)	5.65 ± 0.102	5.19 ± 0.091	0.001^*^	5.71 ± 0.227	5.27 ± 0.121	0.087	5.16 ± 0.142	5.51 ± 0.145	0.129
TG(mmol/L)	2.43 ± 0.084	1.99 ± 0.067	0.000^*^	2.46 ± 0.105	2.02 ± 0.090	0.003^*^	2.35 ± 0.134	1.96 ± 0.107	0.027^*^
TC(mmol/L)	4.55 ± 0.067	4.23 ± 0.064	0.001^*^	4.53 ± 0.083	4.28 ± 0.090	0.004^*^	4.59 ± 0.111	4.19 ± 0.088	0.009^*^
LDL-C(mmol/L)	2.73 ± 0.045	2.56 ± 0.038	0.009^*^	2.75 ± 0.057	2.60 ± 0.061	0.030^*^	2.59 ± 0.074	2.52 ± 0.047	0.008^*^
HDL-C(mmol/L)	1.14 ± 0.031	1.12 ± 0.025	0.636	1.16 ± 0.042	1.12 ± 0.051	0.577	1.11 ± 0.031	1.12 ± 0.028	0.726
VLDL (mmol/L)	0.49 ± 0.017	0.40 ± 0.014	0.000^*^	0.49 ± 0.022	0.40 ± 0.019	0.003^*^	0.47 ± 0.028	0.39 ± 0.028	0.027^*^
DM(%)	143(66.6 %)	72(33.4 %)	0.000^*^	163(74.1 %)	36(25.9 %)	0.000^*^	40(52.6 %)	36(47.4 %)	0.000^*^
EH(%)	221(52.2 %)	201(47.8 %)	0.030^*^	145(60.4 %)	95(39.6 %)	0.024^*^	53(35.3 %)	47(64.7 %)	0.991
Hyperlipidemia(%)	136(54.8 %)	112(45.2 %)	0.000^*^	165(53.9 %)	141(46.1 %)	0.012^*^	79(37.3 %)	133(62.7 %)	0.040^*^
Smoking(%)	287(59.8 %)	193(40.2 %)	0.000^*^	170(57.2 %)	127(42.8 %)	0.000^*^	120(61.9 %)	74(38.1 %)	0.000^*^

*BMI* body mass index, *Glu* glucose, *BUN* blood urea nitrogen, *TG* triglyceride, *TC* total cholesterol, *LDL-C* low density lipoprotein-cholesterol, *HDL-C* high density lipoprotein-cholesterol, *VLDL* very low density lipoprotein, *DM* diabetes mellitus, *EH* essential hypertension. Continuous variable were expressed as mean ± standard error of mean (SEM). *P* value of continuous variables was calculated by independent T-test. The *P* value of categorical variable was calculated by Fisher’s exact test. ^*^ *P* < 0.05

**Table 2:** Characteristics of subjects (Uighur Chinese)

	Total			Male			Female		
	CAD patients	Control subjects	*P* Value	CAD patients	Control subjects	*P* Value	CAD patients	Control subjects	*P* Value
Number(n)	506	351		369	213		137	138	
Age(years)	54.44 ± 0.407	53.37 ± 0.508	0.120	53.94 ± 0.502	53.17 ± 0.617	0.336	53.52 ± 0.710	53.21 ± 0.770	0.220
BMI(kg/m^2^)	27.99 ± 0.183	26.96 ± 0.273	0.001^*^	27.39 ± 0.189	25.39 ± 0.321	0.000^*^	26.63 ± 0.413	25.38 ± 0.406	0.000^*^
Glu(mmol/L)	6.81 ± 0.108	6.35 ± 0.147	0.001^*^	6.73 ± 0.124	6.45 ± 0.159	0.000^*^	6.89 ± 0.218	6.16 ± 0.206	0.004^*^
BUN(mmol/L)	5.68 ± 0.137	6.28 ± 0.281	0.030^*^	5.76 ± 0.171	6.36 ± 0.242	0.030^*^	5.48 ± 0.185	6.14 ± 0.339	0.100
TG(mmol/L)	2.47 ± 0.060	2.19 ± 0.093	0.009^*^	2.46 ± 0.097	2.43 ± 0.080	0.000^*^	2.49 ± 0.133	1.85 ± 0.094	0.000^*^
TC(mmol/L)	4.57 ± 0.101	3.82 ± 0.088	0.000^*^	4.61 ± 0.111	3.84 ± 0.110	0.109	4.51 ± 0.191	3.78 ± 0.126	0.003^*^
LDL-C(mmol/L)	2.77 ± 0.043	2.62 ± 0.050	0.010^*^	2.81 ± 0.048	2.70 ± 0.059	0.000^*^	2.69 ± 0.076	2.50 ± 0.071	0.040^*^
HDL-C(mmol/L)	0.92 ± 0.019	1.22 ± 0.062	0.001^*^	0.91 ± 0.021	1.24 ± 0.045	0.000^*^	0.94 ± 0.033	1.20 ± 0.043	0.010^*^
VLDL (mmol/L)	0.49 ± 0.012	0.44 ± 0.019	0.009^*^	0.49 ± 0.012	0.48 ± 0.027	0.000^*^	0.50 ± 0.027	0.37 ± 0.020	0.000^*^
DM(%)	174(64.2 %)	97(35.8 %)	0.030^*^	119(65.0 %)	64(35.0 %)	0.000^*^	55(62.5 %)	33(37.5 %)	0.000^*^
EH(%)	250(54.1 %)	212(45.9 %)	0.002^*^	162(55.7 %)	129(44.3 %)	0.000^*^	88(51.5 %)	83(48.5 %)	0.535
Hyperlipidemia(%)	262(67.9 %)	212(45.9 %)	0.000^*^	187(68.8 %)	85(35.0 %)	0.000^*^	75(65.8 %)	39(34.2 %)	0.000^*^
Smoking(%)	385(66.7 %)	192(33.2 %)	0.000^*^	309(68.2 %)	144(31.8 %)	0.000^*^	76(61.3 %)	48(38.7 %)	0.001^*^

*BMI* body mass index, *Glu* glucose, *BUN* blood urea nitrogen, *TG* triglyceride, *TC* total cholesterol, *LDL-C* low density lipoprotein-cholesterol, *HDL-C* high density lipoprotein-cholesterol, *VLDL* very low density lipoprotein, *DM* diabetes mellitus, *EH* essential hypertension. Continuous variable were expressed as mean ± standard error of mean (SEM). *P* value of continuous variables was calculated by independent T-test. The *P* value of categorical variable was calculated by Fisher’s exact test. ^*^ *P* < 0.05
2. The distribution of genotypes and alleles of SNP1 (rs12435797), SNP2 (rs2108552), SNP3 (rs1019075) and SNP4 (rs17781919) of Numb gene analysis are shown among Han Chinese and Uighur Chinese in Tables 3 and 4. The distributions of genotypes for four kinds of SNPs were in good agreement with the predicted Hardy-Weinberg equilibrium values (data not shown).
  i. In the total sample of Han Chinese subjects and in Han Chinese males, the overall distribution of SNP2 (rs2108552) genotypes showed a significant difference between CAD and control participants (*P* = 0.013 and *P* = 0.003, respectively) (Table 3). In addition, the dominant model and alleles of SNP2 in the total and male group showed a significant difference between CAD and control subjects (*P* = 0.004 and *P* = 0.001; *P* = 0.007 and *P* = 0.003, respectively). The distribution of the dominant model (CC vs CG + GG) of SNP2 was significantly higher among CAD patients (total: 29.2 %; male: 29.3 %) compared to control subjects (total: 20.5 %; male: 16.4 %). Moreover, C allele frequency of SNP2 was also significantly higher among CAD patients (total: 53.55 %; male: 53.90 %) when compared to control subjects (total: 46.70 %; male: 44.05 %) (Table 3).
  ii. For total Han Chinese, the overall distribution of SNP3 (rs1019075) genotype, recessive, additive model and alleles showed a significant difference between CAD patients and control subjects (*P* = 0.041, *P* = 0.016, *P* = 0.015, and *P* = 0.045, respectively) (Table 3). In addition, the recessive and additive model of SNP3 in males showed a significant difference between CAD patients and control subjects (*P* = 0.030, *P* = 0.020, respectively). The distribution of the recessive model (TT vs CT + CC) of SNP3 was significantly higher in CAD patients (total: 67.3 %; male: 66.8 %) compared to control subjects (total: 59.0 %; male: 56.9 %). Further, C allele frequency of SNP3 was significantly lower in CAD patients (19.1 %) when compared to control subjects (23.3 %) in the total group (Table 3). Among females, there was no significant difference between CAD patients and control subjects with respect to the overall distribution of SNP2 and SNP3 genotypes; dominant and recessive models; and alleles.
  iii. For total, male and female Han Chinese, the overall distribution of SNP1 (rs12435797) and SNP4 (rs17781919) genotypes and allele showed no significant difference between CAD patients and control subjects. (Table 3).
  iv. In the total sample of Uighur Chinese and in Uighur Chinese males, the recessive and additive models of SNP1 showed a significant difference between CAD and control subjects (recessive model: *P* = 0.048 and *P* = 0.034; additive model: *P* = 0.021 and *P* = 0.009, respectively). The distribution of the additive model (GT vs GG + TT) of SNP1 was significantly higher among CAD patients (total: 53.6 %; male: 52.2 %) compared to control subjects (total: 45.4 %; male: 42.5 %). In addition, the overall distribution of SNP1 genotypes and alleles showed no significant difference between CAD patients and control subjects in the total, male and female group. Further, the overall distribution of SNP2, SNP3 and SNP4 genotypes and alleles frequencies showed no significant difference between CAD patients and control subjects in the total, male and female Uighur Chinese (Table 4).

**Table 3:** Genotype and allele distributions in patients with CAD and control subjects (Han Chinese)

				Total			Male			Female		
				CAD n (%)	Control n (%)	*P* value	CAD n (%)	Control n (%)	*P* value	CAD n (%)	Control n (%)	*P* value
rs12435797	Genotype		T/T	173(45.6)	185(43.6)	0.378	125(47.2)	94(43.5)	0.436	48(42.1)	91(43.8)	0.924
(SNP1)			G/G	38(10.1)	56(13.2)		22(8.3)	25(11.6)		16(14.0)	31(14.9)	
			G/T	168(44.3)	183(43.2)		118(44.5)	97(44.9)		50(43.9)	86(41.3)	
		Dominant model	TT	173(45.6)	185(43.6)	0.570	125(47.2)	94(43.5)	0.462	48(42.1)	91(43.8)	0.815
			GT + GG	206(54.4)	239(56.4)		140(52.8)	122(56.5)		66(57.9)	117(56.2)	
		Recessive model	GG	38(10.1)	56(13.2)	0.187	22(8.3)	25(11.6)	0.280	16(14.0)	31(14.9)	0.871
			GT + TT	341(89.9)	368(86.8)		243(91.7)	191(88.4)		98(86.0)	177(85.1)	
		Additive model	GT	168(44.3)	183(43.20)	0.776	118(44.5)	97(44.9)	1	50(43.9)	86(41.3)	0.724
			TT + GG	211(55.7)	241(56.80)		147(55.5)	119(55.1)		64(56.1)	122(58.7)	
	Allele		T	514(67.75)	553(65.20)	0.290	368(69.45)	285(65.95)	0.267	146(64.05)	268(64.45)	0.932
			G	244(32.25)	295(34.80)		162(30.55)	147(34.05)		82(35.95)	148(35.55)	
rs2108552	Genotype		C/C	112(29.2)	88(20.5)	0.013^*^	79(29.3)	36(16.4)	0.003^*^	33(28.9)	52(24.8)	0.724
(SNP2)			G/G	85(22.1)	116(27.1)		58(21.5)	62(28.3)		27(23.7)	54(25.7)	
			C/G	187(48.7)	225(52.4)		133(49.2)	121(55.3)		54(47.4)	104(49.5)	
		Dominant model	CC	112(29.2)	88(20.5)	0.004^*^	79(29.3)	36(16.4)	0.001^*^	33(28.9)	52(24.8)	0.430
			CG + GG	272(70.8)	341(79.5)		191(70.7)	183(83.6)		81(71.1)	158(75.2)	
		Recessive model	GG	85(22.1)	116(27.1)	0.122	58(21.5)	62(28.3)	0.091	27(23.7)	54(25.7)	0.788
			CG + CC	299(77.9)	313(72.9)		212(78.5)	157(71.7)		87(76.3)	156(74.3)	
		Additive model	CG	187(48.7)	225(52.4)	0.293	133(49.2)	121(55.3)	0.203	54(47.4)	104(49.5)	0.728
			CC + GG	197(51.3)	204(47.6)		137(50.7)	98(44.7)		60(52.6)	106(50.5)	
	Allele		C	411(53.55)	401(46.7)	0.007^*^	291(53.9)	193(44.05)	0.003^*^	120(52.6)	208(49.55)	0.460
			G	357(46.45)	457(53.3)		249(46.1)	245(55.95)		108(47.4)	212(50.45)	
rs1019075	Genotype		C/C	21(5.5)	24(5.6)	0.041^*^	12(4.5)	9(4.1)	0.060	9(7.9)	15(7.2)	0.351
(SNP3)			T/T	257(67.3)	252(59.0)		179(66.8)	124(56.9)		78(68.4)	128(61.2)	
			C/T	104(27.2)	151(35.4)		77(28.7)	83(39.0)		27(23.7)	66(31.6)	
		Dominant model	CC	21(5.5)	24(5.6)	1	12(4.5)	9(4.1)	1	9(7.9)	15(7.2)	0.827
			CT + TT	361(94.5)	403(94.4)		256(95.5)	209(95.9)		105(92.1)	194(92.8)	
		Recessive model	TT	257(67.3)	252(59.0)	0.016^*^	179(66.8)	124(56.9)	0.030^*^	78(68.4)	128(61.2)	0.226
			CT + CC	125(32.7)	175(41.0)		89(33.2)	94(43.1)		36(31.6)	81(38.8)	
		Additive model	CT	104(27.2)	151(35.4)	0.015^*^	77(28.7)	83(39.0)	0.020^*^	27(23.7)	66(31.6)	0.158
			CC + TT	278(72.9)	276(64.6)		191(71.3)	133(61.0)		87(76.3)	143(68.4)	
	Allele		C	146(19.1)	199(23.3)	0.045^*^	101(18.85)	101(23.6)	0.081^*^	45(19.75)	96(23.0)	0.371
			T	618(80.9)	655(76.7)		435(81.15)	341(76.4)		183(80.25)	322(77.0)	
rs17781919	Genotype		C/C	384(100.0)	429(99.89)	1	270(100.0)	220(100.0)	1	114(100.0)	209(99.5)	1
(SNP4)			C/T	0(0.0)	1(0.2)		0(0.0)	0(0.0)		0(0.0)	1(0.5)	
	Allele		C	768(100.0)	859(99.9)	1	540(100.0)	440(100.0)	1	228(100.0)	419(99.8)	1
			T	0(0.0)	1(0.1)		0(0.0)	0(0.0)		0(0.0)	1(0.2)	

*CAD* coronary artery disease. The *P* value of genotype was calculated by Fisher’s exact test. ^*^ *P* <0.05

**Table 4:** Genotype and allele distributions in patients with CAD and control subjects (Uighur Chinese)

				Total			Male			Female		
				CAD n (%)	Control n (%)	*P* value	CAD n (%)	Control n (%)	*P* value	CAD n (%)	Control n (%)	*P* value
rs12435797 (SNP1)	Genotype		T/T	92(18.8)	72(20.6)	0.052	70(19.6)	45(21.2)	0.062	22(16.7)	27(19.6)	0.479
			G/G	135(27.6)	119(34.0)		101(28.2)	77(36.3)		34(25.8)	42(30.4)	
			G/T	263(53.6)	159(45.4)		187(52.2)	90(42.5)		76(57.6)	69(50.0)	
		Dominant model	TT	92(18.8)	72(20.6)	0.537	70(19.6)	45(21.2)	0.438	22(16.7)	27(19.6)	1
			GT + GG	398(81.2)	278(79.4)		288(81.4)	167(78.8)		110(83.3)	111(80.4)	
		Recessive model	GG	135(27.6)	119(34.0)	0.048^*^	101(28.2)	77(36.3)	0.034^*^	34(25.8)	42(30.4)	0.371
			GT + TT	355(72.4)	231(66.0)		257(71.8)	135(63.7)		98(74.2)	96(69.6)	
		Additive model	GT	263(53.6)	159(45.4)	0.021^*^	187(52.2)	90(42.5)	0.009^*^	76(57.6)	69(50.0)	0.357
			TT + GG	227(46.4)	191(55.6)		171(47.8)	112(57.5)		56(42.4)	69(50.0)	
	Allele		T	447(45.6)	303(43.3)	0.370	334(45.7)	180(42.45)	0.340	120(45.5)	123(44.6)	0.623
			G	533(54.3)	397(56.7)		389(54.3)	244(57.55)		144(54.5)	153(55.4)	
rs2108552	Genotype		C/C	50(10.2)	31(8.8)	0.556	40(11.2)	20(9.4)	0.596	10(7.5)	11(8.0)	0.613
(SNP2)			G/G	209(42.5)	159(45.6)		154(43.0)	95(44.8)		55(41.0)	64(46.7)	
			C/G	233(47.4)	159(45.6)		164(45.8)	97(45.8)		69(51.5)	62(45.3)	
		Dominant model	CC	50(10.2)	31(8.8)	0.628	40(11.2)	20(9.4)	0.466	10(7.5)	11(8.0)	1
			CG + GG	442(89.8)	318(91.2)		318(88.8)	182(91.6)		114(92.5)	126(92.0)	
		Recessive model	GG	209(42.5)	159(45.6)	0.357	154(43.0)	95(44.8)	0.347	55(41.0)	64(46.7)	0.926
			CG + CC	283(57.5)	190(54.4)		204(57.0)	117(55.2)		79(59.0)	73(53.3)	
		Additive model	CG	233(47.4)	159(45.6)	0.349	164(45.8)	97(45.8)	0.470	69(51.5)	62(45.3)	0.565
			CC + GG	259(52.6)	190(54.4)		194(54.2)	115(54.2)		66(48.5)	75(54.7)	
	Allele		C	333(33.9)	221(31.6)	0.606	244(34.1)	137(32.3)	0.347	89(33.25)	84(30.65)	0.565
			G	651(66.1)	477(68.4)		472(65.9)	287(67.7)		179(66.75)	190(59.35)	
rs1019075	Genotype		C/T	223(45.3)	148(42.5)	0.397	153(42.5)	91(43.1)	0.772	70(53.0)	57(41.6)	0.097
			C/C	66(13.4)	58(16.7)		53(14.7)	35(16.6)		13(9.8)	23(16.8)	
(SNP3)			T/T	203(41.3)	142(40.8)		154(42.8)	85(40.3)		49(37.1)	57(41.6)	
		Dominant model	C/T	223(45.3)	148(42.5)		153(42.5)	91(43.1)		70(53.0)	57(41.6)	
			CC	66(13.4)	58(16.7)	0.191	53(14.7)	35(16.6)	0.460	13(9.8)	23(16.8)	0.098
			CT + TT	426(86.4)	290(83.3)		307(85.3)	176(83.4)		119(90.2)	114(83.2)	
		Recessive model	TT	203(41.3)	142(40.8)	0.895	154(42.8)	85(40.3)	0.587	49(37.1)	57(41.6)	0.724
			CT + CC	289(58.7)	206(59.2)		206(57.2)	126(59.7)		83(62.9)	80(58.4)	
		Additive model	CT	223(45.3)	148(42.5)	0.421	153(42.5)	91(43.1)	1	70(53.0)	57(41.6)	0.132
			CC + TT	269(55.7)	200(57.5)		207(57.5)	161(56.9)		62(47.0)	80(58.4)	
	Allele		C	355(36.05)	264(37.95)	0.438	259(35.95)	261(38.15)	0.394	96(37.3)	103(37.6)	0.612
			T	629(63.95)	432(62.05)		461(64.15)	232(61.85)		168(62.7)	171(62.4)	
rs17781919	Genotype		C/C	476(96.9)	338(96.8)	1	347(96.7)	202(95.7)	0.647	129(97.7)	136(98.6)	0.678
(SNP4)			C/T	15(3.1)	11(3.2)		12(3.3)	9(4.3)		3(2.3)	2(1.4)	
	Allele		C	967(98.45)	687(98.40)	1	706(98.35)	413(97.85)	1	261(98.85)	274(99.3)	0.467
			T	15(1.55)	11(1.60)		12(1.65)	9(2.15)		3(1.15)	2(0.7)	

^*CAD*^ coronary artery disease. The *P* value of genotype was calculated by Fisher’s exact test. ^*^ *P* <0.05.
3. Multiple logistic regression analysis for CAD patients and control subjects from Han Chinese (rs2108552)
  The multivariable logistic regression analysis combining genotypes was conducted for following variables: BMI, Glu TC, TG, LDL-C, DM, EH, hyperlipidemia and smoking, and shown in Table 5. For rs20108552, statistical significance remained after multivariate adjustment in the total and male Han Chinese (total: Odd ratios: 1.687, 95 % confidence interval [CI]: 1.482–1.979, *P* =0.004; male: Odd ratios: 1.498, 95 % confidence interval [CI]: 1.305–1.815, *P* = 0.006) (Table 5).

**Table 5:** Multiple logistic regression analysis for CAD patients and control subjects of Han Chinese (rs2108552)

	Total(*n* = 817)			Male(*n* = 490)			Female(*n* = 327)		
	OR	95 % CI	*P*	OR	95 % CI	*P*	OR	95 % CI	*P*
Dominant model(CC vs CG + GG)	1.687	1.482–1.979	0.004	1.498	1.305–1.815	0.006	1.190	0.507–1.630	0.750
BMI	1.030	0.984–1.079	0.208	1.057	0.993–1.125	0.083	1.021	0.892–1.040	0.340
Glu	1.233	0.945–1.334	0.871	1.175	0.877–1.241	0.477	1.009	0.913–1.116	0.858
TC	1.041	0.886–1.223	0.626	1.007	0.809–1.253	0.951	1.048	0.803–1.368	0.732
TG	1.220	1.079–1.379	0.001	1.291	1.086–1.534	0.004	1.146	0.947–1.386	0.161
LDL-C	1.126	0.907–1.398	0.282	1.276	0.953–1.708	0.101	0.931	0.641–1.353	0.707
DM	2.560	1.811–3.620	0.000	2.318	1.460–3.679	0.000	2.889	1.655–5.078	0.000
EH	1.525	1.121–2.074	0.007	1.675	1.115–2.516	0.013	1.543	0.921–2.887	0.100
Hyperlipidemia	1.450	1.066–1.973	0.030	1.389	1.028–1.879	0.042	1.187	1.104–1.862	0.350
Smoking	1.183	0.868–1.613	0.288	1.148	0.761–1.730	0.511	1.080	0.642–1.814	0.773

**Fig. 2:**
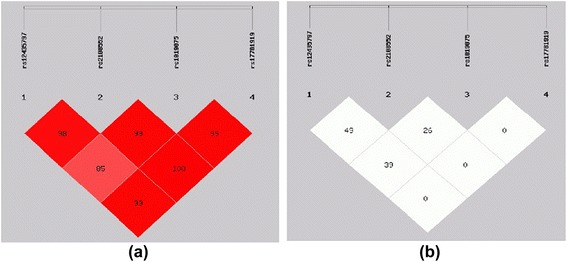
Pairwise estimates of linkage disequilibrium (LD) between each Numb Polymorphism is plotted for Han Chinese using SHEsis platform. Each polymorphism is numbered according to its position in the Numb gene as presented in Fig. 1. (**a**) Showed | D'| and different colors represent different degree of linkage disequilibrium. The darker the Color was the stronger the degree of linkage disequilibrium was (**b**) showed r^2^

**Fig. 3:**
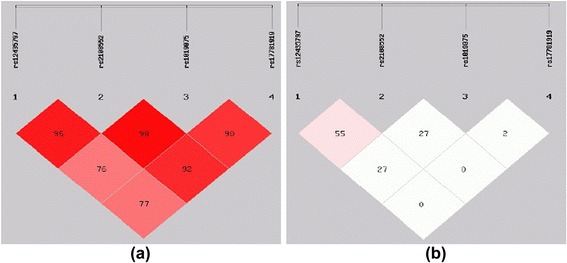
Pairwise estimates of linkage disequilibrium (LD) between each Numb Polymorphism is plotted for Uighur Chinese using SHEsis platform. Each polymorphism is numbered according to its position in the Numb gene as presented in Fig. 1. (**a**) Showed | D'| and different colors represent different degree of linkage disequilibrium. The darker the Color was the stronger the degree of linkage disequilibrium was (**b**) showed r^2^

*EH* essential hypertension, *DM* diabetes mellitus, *CAD* coronary artery disease
4. Haplotypes and linkage disequilibrium
  Haplotypes are established through the use of different combinations of the SNPs in the haplotype-based case–control analysis, independently (Tables 6 and 7) and there are five haplotypes established in all subjects.
  i. In Han Chinese, the frequency of the T-C-T-C haplotype established by SNP1-SNP2-SNP3-SNP4 in these two groups was significantly higher among CAD patients when compared to control subjects (total: OR = 1.334, 95 % CI = 1.096–1.624, *P* = 0.004; male: total: OR = 1.482, 95 % CI = 1.148–1.912, *P* = 0.002). Moreover, the frequency of the G-G-T-C haplotype established by SNP1-SNP2-SNP3-SNP4 in these two groups was also significantly lower among CAD patients when compared to control subjects (total: OR = 0.701, 95 % CI = 0.529–0.929, *P* = 0.013; male: OR = 0.616, 95 % CI =0.432–0.877, *P* =0.007) (Table 6). For females, there was no difference in the frequency of haplotypes between the CAD patients and control subjects. These results of haplotypes were consistent with the results of CC genotype and G allele of SNP2 (rs2108552).
  ii. In Uighur Chinese, the overall distribution of the haplotypes established by SNP1-SNP2-SNP3-SNP4 showed no significant difference between CAD patients and control subjects in the total, male and female group (Table 7).
  iii. The patterns of linkage disequilibrium of the Numb gene among Han and Uighur Chinese are shown in Figs. 2 and 3. All four SNPs are located in one haplotype block, as all of the |D'| values were beyond 0.25 and r^2^ values were below 0.5 We constructed the haplotypes using SNP1, SNP2, SNP3 and SNP4.

**Table 6:** Haplotype analysis in patients with CAD and control subjects (Han Chinese)

			Total(*n* = 817)					Male(*n* = 490)					Female(*n* = 327)				
			Case(freq)	Control(freq)	χ2	*P* value	Odds ratio(95 % CI)	Case(freq)	Control(freq)	χ2	*P* value	Odds ratio(95 % CI)	Case(freq)	Control(freq)	χ2	*P* value	Odds ratio(95 % CI)
1	0100	G G C C	134.72(0.176)	183.90(0.209)	3.375	0.066	0.793(0.619–1.016)	91.93(0.173)	94.94(0.214)	2.500	0.114	0.773(0.561–1.064)	42.77(0.184)	88.94(0.204)	0.449	0.503	0.871(0.580–1.306)
2	0110	G G T C	93.07(0.122)	142.93(0.163)	6.157	0.013^*^	0.701(0.529–0.929)	64.86(0.122)	82.06(0.185)	7.302	0.007^*^	0.616(0.432–0.877)	28.23(0.122)	60.60(0.139)	0.457	0.498	0.848(0.526–1.368)
3	1010	T C T C	407.79(0.534)	400.78(0.456)	8.271	0.004^*^	1.334(1.096–1.624)	285.79(0.537)	196.99(0.444)	9.138	0.002^*^	1.482(1.148–1.912)	122.00(0.526)	204.52(0.469)	1.626	0.202	1.231(0.894–1.694)
4	1100	T G C C	11.27(0.015)	23.24(0.026)	0	0	0	9.06(0.017)	6.06(0.014)	0	0	0	2.23(0.010)	17.20(0.039)	4.853	0.028^*^	0.234(0.057–0.952)
5	1110	T G T C	115.93(0.152)	123.93(0.141)	0.293	0.625	1.071(0.814–1.409)	79.14(0.149)	63.94(0.444)	0.061	0.805	1.046(0.732–1.495)	36.77(0.158)	60.27(0.138)	0.428	0.513	1.160(0.743–1.811)

*CAD* coronary artery disease, *SNP* single nucleotide polymorphism. The *p* value of haplotype was calculated by Fisher’s exact test. ^*^ *p* < 0.05.

Haplotypes with frequencies >0.03 were estimated using SHEsis software; 0 represents major allele and 1 represents minor allele “0100” refers respectively the major allele of the SNP1,minor allele of the SNP2,major allele of the SNP3,major allele of the SNP4

**Table 7:** Haplotype analysis in patients with CAD and control subjects (Uighur Chinese)

			Total(*n* = 857)					Male(*n* = 582)					Female(*n* = 275)				
			Case(freq)	Control(freq)	χ2	*P* value	Odds ratio(95 % CI)	Case(freq)	Control(freq)	χ2	*P* value	Odds ratio(95 % CI)	Case(freq)	Control(freq)	χ2	*P* value	Odds ratio(95 % CI)
1	0100	G G C C	305.11(0.303)	238.23(0.336)	2.284	0.131	0.853(0.693–1.049)	224.57(0.307)	133.44(0.342)	1.548	0.214	0.846(0.650–1.101)	80.43(0.294)	105.09(0.330)	1.082	0.298	0.830(0.584–1.179)
2	0110	G G T C	218.04(0.217)	148.10(0.209)	0.116	0.734	1.042(0.823–1.319)	154.87(0.212)	80.23(0.206)	0.045	0.833	1.033(0.762–1.400)	63.54(0.232)	67.89(0.213)	0.235	0.628	1.101(0.746–1.625)
3	1010	T C T C	333.07(0.331)	215.98(0.305)	1.021	0.273	1.123(0.912–1.383)	245.28(0.335)	118.66(0.304)	1.063	0.302	1.150(0.881–1.501)	87.67(0.320)	96.81(0.304)	0.115	0.735	1.062(0.749–1.508)
4	1100	T G C C	39.770.040)	21.50(0.030)	0.987	0.321	1.310(0.768–2.234)	25.32(0.035)	11.14(0.029)	0.287	0.592	1.216(0.594–2.487)	14.73(0.054)	10.38(0.033)	1.559	0.212	1.669(0.741–3.758)
5	1110	T G T C	88.09(0.088)	66.38(0.094)	0.215	0.643	0.924(0.661–1.291)	64.24(0.088)	36.19(0.093)	0.087	0.769	0.938(0.611–1.439)	23.46(0.086)	30.34(0.095)	0.199	0.656	0.879(0.500–1.548)

*CAD* coronary artery disease, *SNP* single nucleotide polymorphism. The *p* value of haplotype was calculated by Fisher’s exact test. ^*^ *P* < 0.05.

Haplotypes with frequencies >0.03 were estimated using SHEsis software; 0 represents major allele and 1 represents minor allele “0100” refers respectively the major allele of the SNP1,minor allele of the SNP2,major allele of the SNP3,major allele of the SNP4

## Discussion

### Findings

In this case–control study, we have genotyped four kinds of SNPs of Numb gene among Han Chinese and Uighur Chinese, and investigated the association between polymorphism of Numb gene and CAD. We have found that variation in Numb gene is associated with CAD among Han Chinese. This is the first endeavor to study the common allelic variant in Numb gene and it’s association with CAD.

Numb is an important protein for regulating cholesterol absorption and it plays a pivotal role in the development of atherosclerosis. Further, some researches have observed that hypercholesterolemia can cause multiple physiologic outcomes, such as coronary artery disease, diabetes and obesity [39–41]. Therefore, we assume that Numb gene and coronary artery disease might be associated. However, few researches have been conducted regarding the relationship between Numb gene and cardiovascular diseases.

Our study has showed that among Han Chinese, C allele frequency of rs2108552 was higher among male CAD patients when compared to male control subjects. The distribution of the dominant model (CC vs CG + GG) was significantly higher among CAD patients compared to control subjects. The difference remained significant after multivariate adjustment (Table 5). These findings suggest that males carrying the CC genotype of rs2108552 may have a higher risk of CAD

In the total and male Han population, the distributions of the recessive models (TT vs CT + CC) of rs1019075 were significantly higher among CAD patients compared to control subjects. This suggests that people carrying the T allele of rs1019075 may have a higher risk of CAD. T allele frequency of rs1019075 was not higher among CAD patients than control subjects in the total and male group. In logistic regression analysis (TT vs CT + CC), there was no evidence of a statistically significant difference before or after multivariate adjustment (*P* > 0.05, data not shown). A possible explanation is that people carrying T allele have a higher risk from suffering CAD is codetermined by the TT and T allele frequency of rs1019075 in the CAD patients.

We have found that rs2108552 of Numb gene was associated with CAD only in the Han male subgroup. BMI, Glu, TG, TC, LDL-C, prevalence of DM, EH, hyperlipidemia and smoking were higher in male patients in comparison to female CAD patients. Therefore, this indicates the male patients might have higher chance of suffering from cardiovascular disease than the female patients.

In Uighur Chinese subjects, there was no difference between CAD patients and controls with respect to rs2108552, and rs1019075. But another polymorphism of Numb gene, rs12435797, the distribution of the additive model (GT vs GG + TT) was significantly higher among CAD patients in comparison to control subjects in the total population and in the male subgroup. However, statistical significance did not remain after multivariate adjustment (*P* > 0.05). The underlying reason might be ethnic differences which causes different genetic background and life style.

Previous data has shown that SNP4 (rs17781919, G595D) is related to low concentration of LDL-C in humans [29]. In our study, we have neither observed Numb (G595D) variant genotype, nor found significant difference regarding the genotypic distribution of SNP4 (CC and CT) between CAD patients and control subjects among all Han Chinese and Uighur Chinese.

A recent study suggested that statistical method according to the haplotype analysis has more advantages than the individual SNPs analysis to assess complex disease genes, especially there was weak linkage disequilibria between SNPs [42]. The present study is the first haplotype-based case–control endeavor to study the association between the human Numb gene and CAD in Han and Uighur Chinese. In our study, we have found two haplotypes (T-C-T-C and G-G-T-C) of SNP1-SNP2-SNP3-SNP4 in Han male Chinese and assumed that the haplotype (T-C-T-C) is a risk factor for CAD and G-G-T-C is a protective factor for in Chinese Han male population according to the logistic regression and haplotype analyses

### Limitations and shortcomings

The shortcomings of this study are as follows:

1. Because of the limited time, we are only able to conduct a retrospective study. Further, the evidence of this study might be biased due to the unbalanced matching and biased selection. Therefore, a prospective cohort study has to be conducted under a reasonably long time span if one aims to get evidence with higher quality.
2. The representativeness of the study sample might not be sufficient enough due to the limited selection of CAD patients and control subjects. The source of subjects was limited to The First Affiliate Hospital of Xinjiang Medical University, and these subjects may possess some risk factors of cardiovascular disease.

## Conclusions

This is the first scientific endeavor to study the correlation between human Numb gene and CAD among Han Chinese and Uighur Chinese. Findings suggest that rs2108552 may be a novel polymorphism of Numb gene that associates with CAD among male Han Chinese. In addition, CC genotype of rs2108552 and T-C-T-C haplotypes in Numb gene is a possible risk genetic marker for CAD, and G allele and G-T-C-C haplotypes is a possible risk genetic marker for CAD among male Han Chinese which supports the hypothesis that Numb gene variations are involved in the pathogenesis of CAD. These results may broaden the knowledge of genetic variants and disease-association studies. Further studies employing larger sample sizes are required.

## References

[1] Attia YA, Al-Harthi MA, Shiboob MM (2014). Evaluation of quality and nutrient contents of table eggs from different sources in the retail market. Ital J Anim Sci.

[2] Kreisberg RA, Oberman A (2002). Clinical review 141: lipids and atherosclerosis: lessons learned from randomized controlled trials of lipid lowering and other relevant studies. In J Clin Endocrinol Metab.

[3] Writing Group M, Lloyd-Jones D, Adams RJ, Brown TM, Carnethon M, Dai S, De Simone G, Ferguson TB, Ford E, Furie K (2010). Heart disease and stroke statistics--2010 update: a report from the American Heart Association. Circulation..

[4] Lloyd-Jones D, Adams RJ, Brown TM, Carnethon M, Dai S, De Simone G (2010). Executive summary: heart disease and stroke statistics--2010 update: a report from the American Heart Association. Circulation..

[5] Frazier L, Johnson RL, Sparks E (2005). Genomics and cardiovascular disease. J Nurs Scholarsh..

[6] Davis HR, Veltri EP (2007). Zetia: inhibition of Niemann-Pick C1 Like 1 (NPC1L1) to reduce intestinal cholesterol absorption and treat hyperlipidemia. J Atheroscler Thromb..

[7] Davis HR, Compton DS, Hoos L, Tetzloff G (2001). Ezetimibe, a potent cholesterol absorption inhibitor, inhibits the development of atherosclerosis in ApoE knockout mice. Arterioscler Thromb Vasc Biol..

[8] Davis HR, Hoos LM, Tetzloff G, Maguire M, Zhu LJ, Graziano MP, Altmann SW (2007). Deficiency of Niemann-Pick C1 Like 1 prevents atherosclerosis in ApoE−/− mice. Arterioscler Thromb Vasc Biol..

[9] Degirolamo C, Shelness GS, Rudel LL (2009). LDL cholesteryl oleate as a predictor for atherosclerosis: evidence from human and animal studies on dietary fat. J Lipid Res.

[10] Altmann SW, Davis HR, Zhu LJ, Yao X, Hoos LM, Tetzloff G (2004). Niemann-Pick C1 Like 1 protein is critical for intestinal cholesterol absorption. Science..

[11] Wang LJ, Wang J, Li N, Ge L, Li BL, Song BL (2011). Molecular characterization of the NPC1L1 variants identified from cholesterol low absorbers. J Biol Chem..

[12] Berge KE, Tian H, Graf GA, Yu L, Grishin NV, Schultz J, Kwiterovich P, Shan B, Barnes R, Hobbs HH (2000). Accumulation of dietary cholesterol in sitosterolemia caused by mutations in adjacent ABC transporters. Science..

[13] Lee MH, Lu K, Hazard S, Yu H, Shulenin S, Hidaka H (2001). Identification of a gene, ABCG5, important in the regulation of dietary cholesterol absorption. Nat Genet..

[14] Yu L, Hammer RE, Li-Hawkins J, Von Bergmann K, Lutjohann D, Cohen JC (2002). Disruption of Abcg5 and Abcg8 in mice reveals their crucial role in biliary cholesterol secretion. Proc Natl Acad Sci U S A..

[15] Yu L, Li-Hawkins J, Hammer RE, Berge KE, Horton JD, Cohen JC (2002). Overexpression of ABCG5 and ABCG8 promotes biliary cholesterol secretion and reduces fractional absorption of dietary cholesterol. J Clin Invest..

[16] Rudel LL, Lee RG, Parini P (2005). ACAT2 is a target for treatment of coronary heart disease associated with hypercholesterolemia. Arterioscler Thromb Vasc Biol.

[17] Joyce C, Skinner K, Anderson RA, Rudel LL (1999). Acyl-coenzyme A:cholesteryl acyltransferase 2. Curr Opin Lipidol..

[18] Ge L, Wang J, Qi W, Miao HH, Cao J, Qu YX (2008). The cholesterol absorption inhibitor ezetimibe acts by blocking the sterol-induced internalization of NPC1L1. Cell Metab..

[19] Wang LJ, Song BL (1821). Niemann-Pick C1-Like 1 and cholesterol uptake. Biochim Biophys Acta..

[20] Li PS, Fu ZY, Zhang YY, Zhang JH, Xu CQ, Ma YT (2014). The clathrin adaptor Numb regulates intestinal cholesterol absorption through dynamic interaction with NPC1L1. Nat Med..

[21] Roncarati R, Sestan N, Scheinfeld MH, Berechid BE, Lopez PA, Meucci O (2002). The gamma-secretase-generated intracellular domain of beta-amyloid precursor protein binds Numb and inhibits Notch signaling. Proc Natl Acad Sci U S A..

[22] Dho SE, Jacob S, Wolting CD, French MB, Rohrschneider LR, McGlade CJ (1998). The mammalian numb phosphotyrosine-binding domain. Characterization of binding specificity and identification of a novel PDZ domain-containing numb binding protein, LNX. J Biol Chem.

[23] Nishimura T, Kaibuchi K (2007). Numb controls integrin endocytosis for directional cell migration with aPKC and PAR-3. Dev Cell..

[24] Tong X, Zitserman D, Serebriiskii I, Andrake M, Dunbrack R, Roegiers F (2010). Numb independently antagonizes Sanpodo membrane targeting and Notch signaling in Drosophila sensory organ precursor cells. Mol Biol Cell..

[25] Verdi JM, Bashirullah A, Goldhawk DE, Kubu CJ, Jamali M, Meakin SO (1999). Distinct human NUMB isoforms regulate differentiation vs. proliferation in the neuronal lineage. Proc Natl Acad Sci U S A.

[26] Zwahlen C, Li SC, Kay LE, Pawson T, Forman-Kay JD (2000). Multiple modes of peptide recognition by the PTB domain of the cell fate determinant Numb. EMBO J..

[27] Li SC, Zwahlen C, Vincent SJ, McGlade CJ, Kay LE, Pawson T (1998). Structure of a Numb PTB domain-peptide complex suggests a basis for diverse binding specificity. Nat Struct Biol..

[28] Li SC, Songyang Z, Vincent SJ, Zwahlen C, Wiley S, Cantley L (1997). High-affinity binding of the Drosophila Numb phosphotyrosine-binding domain to peptides containing a Gly-Pro-(p)Tyr motif. Proc Natl Acad Sci U S A..

[29] Wei J, Fu ZY, Li PS, Miao HH, Li BL, Ma YT (2014). The clathrin adaptor proteins ARH, Dab2, and numb play distinct roles in Niemann-Pick C1-Like 1 versus low density lipoprotein receptor-mediated cholesterol uptake. J Biol Chem..

[30] Li X, Ma YT, Xie X, Yang YN, Ma X, Zheng YY (2014). Association of Egr3 genetic polymorphisms and coronary artery disease in the Uygur and Han of China. Lipids Health Dis..

[31] Zhu Q, Fu Z, Ma Y, Yang H, Huang D, Xie X (2013). A novel polymorphism of the CYP2J2 gene is associated with coronary artery disease in Uygur population in China. Clin Biochem.

[32] Pan S, Nakayama T, Sato N, Izumi Y, Soma M, Aoi N (2013). A haplotype of the GOSR2 gene is associated with essential hypertension in Japanese men. Clin Biochem.

[33] Patel S, Flyvbjerg A, Kozakova M, Frystyk J, Ibrahim IM, Petrie JR (2008). Variation in the ADIPOQ gene promoter is associated with carotid intima media thickness independent of plasma adiponectin levels in healthy subjects. Eur Heart J.

[34] Zheng YY, Xie X, Ma YT, Yang YN, Fu ZY, Li XM (2011). Relationship between a novel polymorphism of the C5L2 gene and coronary artery disease. PLoS One..

[35] Xie X, Ma YT, Fu ZY, Yang YN, Ma X, Chen BD (2009). Association of polymorphisms of PTGS2 and CYP8A1 with myocardial infarction. Clin Chem Lab Med..

[36] Friedewald WT, Levy RI, Fredrickson DS (1972). Estimation of the concentration of low-density lipoprotein cholesterol in plasma, without use of the preparative ultracentrifuge. Clin Chem.

[37] Nakayama T, Soma M, Rahmutula D, Ozawa Y, Kanmatsuse K (2001). Isolation of the 5′-flanking region of genes by thermal asymmetric interlaced polymerase chain reaction. Med Sci Monit.

[38] Shi YY, He L (2005). SHEsis, a powerful software platform for analyses of linkage disequilibrium, haplotype construction, and genetic association at polymorphism loci. Cell Res.

[39] Shoelson SE, Lee J, Goldfine AB (2006). Inflammation and insulin resistance. J Clin Invest..

[40] Guilherme A, Virbasius JV, Puri V, Czech MP (2008). Adipocyte dysfunctions linking obesity to insulin resistance and type 2 diabetes. Nat Rev Mol Cell Biol..

[41] Bluher M (2009). Adipose tissue dysfunction in obesity. Exp Clin Endocrinol Diabetes..

[42] Morris RW, Kaplan NL (2002). On the advantage of haplotype analysis in the presence of multiple disease susceptibility alleles. Genet Epidemiol.

